# Prenatal, birth and early life predictors of sedentary behavior in young people: a systematic review

**DOI:** 10.1186/s12966-016-0389-3

**Published:** 2016-06-07

**Authors:** Maria Hildebrand, Guro P. Øglund, Jonathan C. Wells, Ulf Ekelund

**Affiliations:** The Department of Sports Medicine, Norwegian School of Sport Sciences, P.O Box 4014, Ullevål Stadion, 0806 Oslo, Norway; Childhood Nutrition Research Centre, UCL Institute of Child Health, London, UK; Medical Research Council Epidemiology Unit, University of Cambridge, Cambridge, UK

**Keywords:** Sedentary behavior, Youth, Children, Determinants, Early life, Prenatal

## Abstract

**Background:**

Our aim was to systematically summarize the evidence on whether prenatal, birth and early life factors up to 6 years of age predict sedentary behavior in young people (≤18 years).

**Methods:**

PRISMA guidelines were followed, and searches were conducted in PubMed, SPORTDiscus, EMBASE and Web of Science up to December 1, 2015. We included observational (non-intervention) and longitudinal studies, that reported data on the association between one or more of the potential predictors and objectively or subjectively measured sedentary behavior. Study quality was assessed using a formal checklist and data extraction was performed using standardized forms independently by two researchers.

**Results:**

More than 18,000 articles were screened, and 16 studies, examining 10 different predictors, were included. Study quality was variable (0.36-0.95). Two studies suggest that heritability and BMI in children aged 2–6 years were significant predictors of sedentary behavior later in life, while four and seven studies suggest no evidence for an association between gestational age, birth weight and sedentary behavior respectively. There was insufficient evidence whether other prenatal, birth and early life factors act as predictors of later sedentary behavior in young people.

**Conclusion:**

The results suggest that heritability and early childhood BMI may predict sedentary behavior in young people. However, small number of studies included and methodological limitations, including subjective and poorly validated sedentary behavior assessment, limits the conclusions.

**Trial registration:**

The systematic review is registered in the International Prospective Register of Systematic Reviews, PROSPERO, 17.10.2014 (CRD42014014156).

**Electronic supplementary material:**

The online version of this article (doi:10.1186/s12966-016-0389-3) contains supplementary material, which is available to authorized users.

## Background

Sedentary behavior, defined as a distinct class of waking behavior in a seated or reclining posture that requires an energy expenditure ≤1.5 METs [1], is highly prevalent in contemporary youth [2–4]. For example, studies using objective measures of sedentary time estimate that 41 to 78 % of awake time is spent sedentary in young people aged 7–15 years old [5]. Further, high amounts of sedentary behavior may be associated with adverse health outcomes [6–9], and to be able to implement effective interventions and inform policy, increased knowledge about predictors and determinants of sedentary behavior are needed. Previous systematic reviews have mainly focused on environmental, social, behavioral and policy factors during childhood and adolescence (>6 years of age) as determinants of later sedentary behavior [10, 11]. However, studies have shown that high amounts of sedentary time are present already in younger children (3–5 years of age) [12], that this behavior increases during childhood [13, 14] and tracks from childhood to adolescent and adulthood [15], suggesting that important factors associated with sedentary behavior may manifest early in life, perhaps already during the fetal period or at birth.

According to the Developmental Origins of Health and Disease hypothesis, non-optimal growth and environmental conditions during fetal life and early childhood may result in permanent changes in the body’s structure, function and metabolism [16]. These adaptations, potentially caused by epigenetics [16] and irreversible, may lead to increased risk of diseases and an altered behavior later in life. For example, birth weight, which is used as a marker of intrauterine growth and the intra-uterine environment, is broadly inversely associated with the risk of cardiovascular disease [17, 18], type 2 diabetes [19, 20], and all-cause mortality [21, 22]. Furthermore, results from animal studies suggest that the offspring of undernourished mothers are less active and more sedentary compared with normal offspring [23, 24], and the underlying mechanism for this association might be due to remodeling of the hypothalamus through alterations in availability of nutrients or hormonal signaling [23]. Another possible hypothetical pathway between prenatal, birth and early life factors, that are usually categorized as physical factors [25], and sedentary behavior might be through excessive adiposity tissue. High and low birth weights [26–30], genetics [31], maternal physical activity during pregnancy [32] and early rapid weight gain [33–35] are all predictors of later obesity, which might constrain physical movement [36] and lead to a sedentary lifestyle [37–39]. Moreover, these putative underlying physical factors acting during gestation, at birth and in early life may, directly or indirectly, predict sedentary behavior through a variety of other biological mechanisms, including reduced aerobic fitness [40], lower muscle strength [41], decreased lung function [42] and genetic abnormality [43].

Therefore, the aim of this study was to examine whether prenatal, birth and early life physical factors (up to 3–6 years of age) are predictors of sedentary behavior by synthesizing the evidence from observational research in young people ≤ 18 years old.

## Methods

### Study inclusion criteria

The review is registered in PROSPERO CRD42014014156, and follows the PRISMA guidelines. The review aimed to identify all observational (non-intervention) longitudinal studies (prospective and retrospective) reporting data on the association between one or more of the potential predictors and sedentary behavior in young people (aged ≤18 years). Only studies that examined factors which may be causally associated with the outcome (factors that precedes sedentary behavior later in life), rather than correlates (factors which are statistically associated with the outcome in cross-sectional analyses), were included. The term "determinant" is often used in similar studies [10, 11], however since evidence from observational studies does not prove cause-and-effect relationship [44], we here use the term "predictor".

**Fig. 1:**
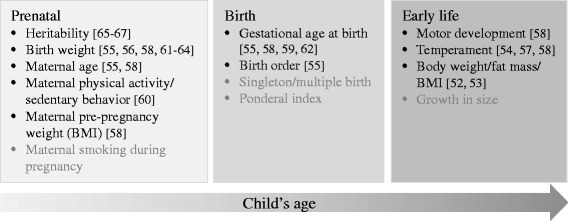
Potential prenatal, birth and early life predictors of sedentary behavior included in the current review. References of included studies in squared brackets

We adopted the following inclusion criteria: (i) written in English (ii) published after 01/01/2000; (iii) published as journal articles or reports; and (iv) including healthy children. Thus, studies only including a specific group (e.g., only obese or children with premature birth) were excluded from this review. The potential predictors were identified as prenatal, birth and early life characteristics, previously classified under the physical domain [25, 45] when studied in relation to physical activity [46, 47] (Fig. 1). We have defined early life from birth to three years of age since motor development up to three years of age is characterised by achieving fundamental developmental milestones, e.g., sit with and without support, supported and unsupported standing and walking [48], while temperament, referring to biologically based individual differences in emotional, motor, and attentional reactivity, may interact with the environment over time [49]. To take into account potentially critical periods such as the adiposity rebound [50] when considering growth and body size (body weight/fat mass/body mass index, BMI), we included studies examining these factors between birth and 6 years of age. In addition, gene variants may influence on the in utero development [31], and was therefore explored as a potential predictor.

Sedentary behavior includes activities such as watching television, using a computer or sitting at school. Studies were included if they measured total sedentary time (e.g., minutes/day) or a specific type of sedentary behaviors (e.g., TV-viewing, computer use etc.), measured either objectively (e.g., with an accelerometer) or subjectively (e.g., with self- or parentally reported questionnaires).

### Search strategy

**Fig. 2:**
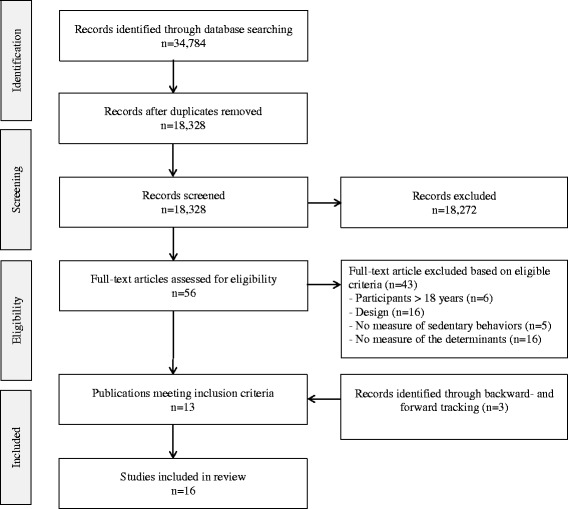
Flow diagram of review process

Two researchers performed a systematic literature search in the electronic databases PubMed, SPORTDiscus, EMBASE and Web of Science including studies published between January 2000 and December 1, 2015 (Fig. 2). The searches included terms related to sedentary behavior (sedentary time, TV-viewing, etc.) in combination with the sample of interest (children, youth, adolescent etc.) and terms related to the potential predictors (birth weight, motor development etc.). An additional file shows a detailed overview of the search strategy [see Additional file 1].

Identified articles were imported to Reference Manager Professional Edition (version 12, Thomson Reuters, San Francisco, CA, USA) and duplicates were removed. One researcher screened the titles, whereas two researchers independently screened all abstracts to minimize the risk of elimination of eligible studies by mistake. If any doubts the articles were included to the next phase. Two researchers independently performed the full-text review. The reference lists of all included studies were reviewed (backward tracking), and a citation search was performed in the database Web of Science (forward tracking). In addition, all reviewers manually searched through personal reference databases.

### Data extraction and statistical analysis

Data were extracted using standardized forms independently by two researchers. Any disagreements were resolved by consensus or by a third researcher. We extracted the following data; study characteristics (title, author, year, study design, country, number of participants, subject characteristics, year of baseline measure and year of follow-up), predictors examined and assessment method, sedentary behavior and assessment method, statistics and analysis, main results and results stratified by sub-groups if provided in the article (e.g., sex and age-groups). Researchers were not blinded to the authors or journals when extracting data.

The primary aim was to synthesize the evidence by formal meta-analyses on the association between the predictors and sedentary behavior. However due to few studies retrieved, and heterogeneity in the exposure and outcome measures in these studies, this was not possible. Therefore, the data were synthesized narratively.

### Quality assessment

Quality assessment of the included studies was performed using a formal checklist [51]. Two independent researchers performed the quality assessment, and any disagreements where resolved by consensus or by consultation with a third researcher if necessary. Studies were given scores (0- No, 1- Partial, 2-Yes) on 11 items based on the degree to which the criteria were met [Additional file 2]. For each study, a summary score was calculated as the sum of scores from each item divided by the highest possible score. Study quality scores therefore ranged from 0–1, where a higher score corresponds to higher quality. The result of the quality assessment was used for discussion of the quality of the studies and no study was excluded based on this assessment.

## Results

The database searches resulted in more than 34,000 potentially relevant articles, but after removal of duplicates approximately 18,300 articles remained. Details of the search and screening process are shown in Fig. 2. The title and abstract review resulted in the retrieval of 56 full-text articles, which were reviewed in detail. Of these, 13 studies met the criteria for study inclusion. The backward- and forward tracking process of the included studies resulted in additional three identified studies meeting our inclusion criteria. In total, 16 unique studies including ten potential predictors were included (Fig. 1). Individual study characteristics, in addition to the main results showing the association between the predictors and sedentary behavior are presented in Table 1.

**Table 1:** Individual study characteristics and results showing the relation (and direction) between the included predictors and sedentary behavior in young people

First author (year)	Country	Age, baseline (years)	Mean age (SD), follow-up (years)	n (% girls)	Assessment of sedentary behavior	Predictors and association with sedentary behavior										Quality assessment
						Heritability	Maternal age	Maternal PA	Maternal pre-pregnancy weight	Birth weight	Gestational age	Birth order	Motor development	Temperament	BMI	
Fisher (2010) [66]	UK		11.2 (0.5)	234 (54)	Acc	0										0.91
Haberstick (2014) [65]	USA		15.1 (2.2)	2,847 (52)	Self-report^a^	+										0.77
van der Aa (2012) [67]	NED		15.9 (1.6)	6,011 (56)	Self-report SCT	+										0.86
Pearce (2012) [55]	UK	0	8-10 (range)	482 (52)	Acc		0			0	0	0				0.73
Wijtzes (2013) [58]	NED	0-1	2.1 (0.1)	347 (48)	Acc		0		0	0	0		0	0		0.82
Pivarnik (2014) [60]	USA		8-10 (range)	20	Acc			0								0.36
Byun (2011) [61]	USA		4.3 (0.6)	331 (49)	Acc					0						0.95
Gopinath (2013) [56]	AUS		12.7 (0.4) 17-18 (range)	1,794 (50) 752 (53)	Self-report SCT					0						0.82
Hildebrand (2015) [63]	ICAD^b^		6-18 (range)	10,793 (53)	Acc					0						0.95
Peneau (2011) [62]	FRA		7-9 (range)	2,207 (49)	Parent-report TV					0	0					0.82
Ridgway (2011) [64]	Overall^c^ EYHS^d^ UK UK BRA	0	12.0 (2.9) 14.5 (0.5) 10.2 (0.3) 13.3 (0.3)	4170 1,240 (53) 811 (56) 1,647 (56) 472 (48)	Acc Acc Acc Acc					0 0 - 0 0						0.95
Lowe (2015) [59]	UK	0	11 15	5327 (52) 1947 (55)	Acc						0					0.86
Thompson (2013) [54]	USA	3m-1	1.5	110-217	Parent-report TV									+/0		0.86
Radesky (2014) [57]	USA	9m	2.0	7450 (49)	Parent-report TV									+		0.91
Fuller-Tyszkiewicz (2012) [52]	AUS	2.3 6.3	4.3 (0.4) 6.3 (0.5) 8.3 (0.4) 10.3 (0.5)	4,724 (49) 4,340 (49)	Parent-report TV										+	0.64
Hands (2011) [53]	AUS	6	8.1 (0.4)	1,271 (49)	Parent-report TV										+	0.77

0, no association; +, positive association; -, negative association

*Acc* accelerometer, *AUS* Australia, *BRA* Brazil, *FRA* France, *M* months, *NED* Netherlands, *PA* physical activity, *TV* time TV-viewing, *SCT* screen time, *R* retrospectively

^a^ Include hours watching TV, sitting doing nothing and sitting listening to music per week. ^b^Data from eight studies in the International Children’s Accelerometry Database (ICAD) collected in United Kingdom, Denmark, Estonia, Norway, Portugal, Switzerland and Brazil. ^c^Data presented as overall from the meta-analysis and for each study included in the meta-analysis. ^d^Data from the European Youth Heart Study (EYHS) collected in Norway, Portugal, Estonia and Denmark

### Study characteristics

Of the 16 included studies, eight were longitudinal prospective birth cohorts [52–59], while three studies had retrospective data collection [60–62], and two studies included a combination of both prospective and retrospective measures [63, 64]. Three studies examining heritability of sedentary behavior were cross-sectional twin studies [65, 66] or twin-family studies (i.e., including both twins and a non-twin siblings) [67]. The majority of the studies were conducted in the USA (*n* = 5) [54, 57, 60, 61, 65], UK (*n* = 5) [55, 59, 63, 64, 66], or Australia (*n* = 3) [52, 53, 56]. All studies were published from 2010 and onwards, with five studies being published during 2014 and 2015 [57, 59, 60, 63, 65]. The sample sizes ranged from 20 [60] to 10,793 participants [63]. Eight studies measured sedentary time objectively [55, 58–61, 63, 64, 66], while the remaining studies used subjective methods, including self-reported screen time [56, 67], parent-reported TV-time [52–54, 57, 62], or a summary of time spent watching TV, sitting doing nothing and sitting listening to music [65]. The included age groups at follow-up were 0–6 years (*n* = 4) [54, 57, 58, 61], 7–12 years (*n* = 5) [52, 55, 60, 62, 66], or a combination of different age groups ≤18 years (*n* = 7) [53, 56, 59, 63–65, 67].

## Quality assessment

The included articles had a quality score between 0.36 and 0.95 (range 0 – 1) (Table 1), and 11 studies had a score above 0.80. The most common limitation was the use of a subjective and poorly validated measure of the outcome (*n* = 8), such as parentally reported TV-viewing. Other limitations include incomplete description of participant selection (*n* = 8), incomplete participant characteristics (*n* = 6), variance estimates not provided for all results (*n* = 7), lack of controlling for several confounding variables (*n* = 5) and insufficient reporting of results (*n* = 4).

### Prenatal predictors and sedentary behavior

No studies were identified that examined whether maternal smoking or maternal sedentary behavior during pregnancy act as predictors of sedentary behavior in the offspring. Based on a limited number of studies, there was no evidence for an association between maternal pre-pregnancy weight [58] and maternal physical activity during pregnancy [60] and objectively measured sedentary time in children aged 2 and 8–10 years. Similarly, no association between maternal age at birth and objectively measured sedentary time in children aged 2 or 8–10 years [55, 58] was observed.

Two studies found that heritability was a significant contributor on self-reported leisure sedentary time/screen time in children aged 12 years or older [65, 67]. One of these studies reported higher heritability in girls (girls versus boys: 30 % versus 9 %) [65], while another reported the opposite (19 % versus 35 %) [67]. Finally, one study observed a borderline none significant heritability effect on the variance in objectively measured sedentary time in 9-12-year-old children [66].

Seven studies examined the association between birth weight and sedentary behavior. Based on five studies, there is no evidence for an associations between birth weight and objectively measured sedentary time [55, 58, 61], total recreational screen time [56] or increased risk of TV-viewing ≥ 2 h per day [62]. One study presented data using a combined meta-analysis from four cohorts, and observed no evidence for an association between birth weight and objectively measured sedentary time [64]. However in study specific analyses, a low birth weight was associated with higher amounts of sedentary time in one of the studies (the Roots-study, *n* = 811), whereas a high birth weight was associated with higher amounts of sedentary time in another (The Pelotas Birth Cohort, *n* = 472). The latter study was the only study in which gestational age was assessed, and after adjusting for this covariate, the positive association was no longer significant [64]. Finally, the seventh study used pooled data from eight studies (*n* = 10,793) and found that high birth weight was associated with greater amount of time spent sedentary, however this association was partly mediated by waist circumference [63].

### Birth predictors and sedentary behavior

We did not identify any study examining whether ponderal index at birth was associated with subsequent sedentary behavior. One study found no evidence for an association between birth order and objectively measured sedentary time in 8 to 10-year-olds [55].

There was no evidence for an association between gestational age and objectively measured sedentary time in 8 to 10-year-olds [55], or TV-viewing ≥ 2 h per day in 7-9-year-olds [62]. In addition, preterm birth (<37 weeks gestation) was not associated with increased sedentary time in children aged 2, 11 and 15 years in comparison with full term birth [58, 59].

### Early life predictors and sedentary behavior

We did not identify any study examining whether infant and childhood growth patterns predict later sedentary behavior, however two studies examined the association between BMI and later sedentary behavior. It appears that BMI measured in children aged 2–6 years old positively predicts TV-viewing two or several years later [52, 53], however dietary intake mediated the relationship for the older children in one of the studies [52].

Inconsistent evidence was observed for the association between early life temperament and sedentary behavior. Among infants and toddlers, two studies found a positive association between crying duration (hours/day) [54] and having problems with self-regulation (i.e., sleep, mood and behavior regulation and attention) [57] and viewing TV/video. In contrast, no association was found between two other dimensions of infant and toddlerhood temperament (i.e., activity level such as arm and leg movements, squirming etc. and fussiness) and objectively measured sedentary time/TV-exposure in children aged 1.5-2 years [54, 58].

One study showed no association between having a delayed gross motor development at 1 year and sedentary time in 2-year-old children [58].

## Discussion

We have systematically summarized the existing knowledge on potential prenatal, birth and early life predictors of sedentary behavior in young people. Few studies have examined whether these factors act as predictors of sedentary behavior later in life. However, the results suggest that heritability and childhood body weight (≤6 years) may be possible predictors of later sedentary behavior, while birth weight and gestational age are unlikely important predictors of this behavior.

### Prenatal factors

Maternal age at birth, maternal pre-pregnancy weight and maternal physical activity during pregnancy were not related to sedentary behavior in the offspring [55, 58, 60]. However, it is difficult to distinguish between the potential biological effects that may occur during fetal life due to maternal age (e.g., young mothers who are still growing might be competing for nutrients with the fetus, or higher maternal age could influence genetic abnormality [43]), and other non-biological differences later in life (e.g., behavior, education, socioeconomic status). Due to the low number of studies, of which one was categorized as low quality, it is not possible to draw any firm conclusions of whether maternal factors during pregnancy may influence later sedentary behavior in their offspring. Additional studies including high quality, objective measures of physical activity and sedentary time in women before and during pregnancy are needed to examine whether these behaviors may transmit to their offspring.

Data from twin studies comparing differences in agreement between monozygotic and dizygotic twins are useful to estimate heritability or the genetic contribution to a given trait, e.g., sedentary behavior. If a monozygotic twin pair is more similar than a dizygotic twin pair, this suggests heritability, whereas the remaining variance is due to environmental influences [68]. Two studies suggest heritability of variation in self-reported sedentary behavior, however one study reported higher heritability among girls [65], and the other among boys. This difference may be explained by different definitions of sedentary behavior. While one study included time spent on computer and video games [67], which may be more common activities in adolescent boys than girls, this was not included in the other study [65]. The third study examining whether heritability influenced sedentary behavior found a borderline-significant association with objectively measured sedentary time in younger children [66], however a small sample size, and a younger age group (9–12 years) may explain the non-significant associations. It can be assumed that younger children are more influenced by non-heritable factors such as parents and the school environment than older children are. This hypothesis is supported by one study showing an increased genetic contribution with increased age [67], and further supported by studies in adults in which the heritability of sedentary behaviors appears greater in magnitude than in young people (>30 %) [69–71]. Additional studies are needed to identify regions within a genome contributing to variation in sedentary behavior [71]. While no robust genetic markers for this behavior have been identified through genome wide association studies, a linkage between objectively measured sedentary time, and two markers (D18S1102, D18S64) on chromosome 18 in overweight and obese youth has been observed [72].

The possible mechanisms for an association between birth weight and subsequent sedentary behavior are not clear. However a low birth weight is associated with lower muscle mass, strength [41, 73] and aerobic fitness later in life [40, 74], and both low and high birth weights are associated with several measures of adiposity [26–30]; factors that may be related to sedentary behavior. In adjusted analysis, only one study observed a positive association between birth weight and sedentary time, however this was partly mediated by abdominal adiposity [63]. The remaining six studies found no evidence for a relationship with objectively [55, 58, 61, 64] and subjectively measured sedentary behavior [56, 62]. Although five studies used an objective measure for sedentary time, a formal meta-analysis was not possible due to several reasons. First, sedentary time was expressed in diverse metrics (e.g., % sedentary time vs. minutes of sedentary time per day) and different thresholds were used to define time spent sedentary. Secondly, one study [63] is considerably larger compared to the others (Table 1) and would substantially influence the result of a formal meta-analysis. Finally, a meta-analysis of few studies with low methodological quality and heterogeneity in study design, participants and measurements is not recommended since it can lead to misleading results and interpretations [75]. Based on the results from the available literature, birth weight is not an important predictor for sedentary behavior in children and youth, and if such association is observed it may be explained by a positive association between higher birth weight and adiposity [63]. These observations are in agreement with a recent meta-analysis in children and youth, on the association between birth weight and physical activity [76].

### Factors related to birth

Previous studies suggest that being born preterm is associated with decreased lung function, which persists as a degree of functional impairment through life [42, 77]. Therefore, children born preterm might be more sedentary compared to children born at term. We identified four studies all suggesting that gestational age is not associated with sedentary behavior in young people [55, 58, 59, 62], despite the fact that one study showed that preterm-born children had lung function deficits earlier in childhood [59]. The results are further supported by studies showing no association between preterm birth and objectively measured physical activity in children [59] and adults [78]. Children born preterm are often encouraged to be physically active, in order to promote their health. This may therefore negate any tendency for preterm children to be less active than their term born peers.

### Early childhood factors

Early motor development has been associated with higher physical activity in childhood [47, 76] and it is plausible that infants and children who experience later or impaired motor development automatically choose to be more sedentary. Higher motor coordination (i.e., ball throwing, one-foot balance and walking backwards) at age 10 years have been associated with less screen time in adolescence and adulthood [79]. However, we did only identify one, relatively small study, suggesting no association between a delayed early life motor development and objectively measured sedentary time in toddlers [58]. Therefore, studies with larger sample sizes and longer duration of follow-up are warranted to examine whether impaired motor development acts as a predictor of sedentary behavior, and whether this association is modifiable [80].

Infant temperament has been associated with the risk for development of overweight and obesity in children [81] and it has been suggested that infants and toddlers scoring higher on selected dimensions of temperament (e.g., sad, aggressive, active) are more likely to be given an obesogenic diet by their caregivers [82–85]. It is also plausible that the TV can be used to sooth and entertain children who are perceived as more aggressive and difficult to calm. Two studies suggested both positive [54, 57] and no association [54] between early life temperament and parent-reported TV time, and the latter is supported by one study using objectively measured sedentary behavior [58]. Explanation for the mixed results may be explained by the assessment of different dimensions of infant temperament, and diversity between studies. The studies using parent-reported TV time suggest that the associations were stronger among mothers with low socioeconomic status [54, 57], and in overweight or obese mothers [54]. Hence, it seems as strategies aimed at educating low income and often overweight mothers in other ways to cope with challenging temperament traits in their children rather than using the TV, may be an important intervention to reduce the development of not only sedentary behavior, but also overweight and obesity among these children.

Both infancy and childhood rapid weight gain are independent risk factors for later obesity [34, 86], and possibly predictors of sedentary behavior since higher adiposity at one point appear to predict sedentary time later in childhood [87, 88]. While infant adiposity has been associated with lower activity level later in infancy [89], we did not identify any study examining the association between early rapid weight gain and sedentary behavior. However, two studies suggested that a higher BMI in 2-6-year-olds was associated with greater time spent sedentary later in life [52, 53]. This association is also observed longitudinally in older children [37], and supports the notion that sedentary behavior may be the result of overweight and obesity. However, the reason why higher levels of adiposity may predict higher amounts of sedentary time is not known. Explanations such as musculoskeletal pain [36], negative body image [90], bullying [90], and physiological limitations including impaired mitochondrial function [91] and insulin resistance [92] have been suggested, but further research is needed to obtain a better understanding of the underlying mechanisms.

### Methodological issues

Strengths of this review included a comprehensive search strategy, the use of a standardized protocol, an up to date search including papers published until December 2015, and the inclusion of several potential predictors for sedentary behavior. As with any systematic review the methodological quality is no better than the studies included in the review. The main limitations with the review are the small number of retrieved studies, heterogeneous data and methodological quality in the included studies. Despite the large number of high quality birth cohorts available globally, few have included measures of sedentary behavior aimed at examining early life predictors of these behaviors. Eight out of 16 studies included in this review assessed sedentary time objectively by accelerometry. While a hip-placed accelerometer can provide sedentary data over a prolonged period, they are less valid in distinguishing sedentary postures, such as lying or sitting, from other light-intensity activities performed while standing [93]. In addition, different definitions of sedentary time and different data reductions methods may explain some of the dissimilarity in the results. Furthermore, the variability in time spent sedentary in children and adolescents is large, and only few days of measurement may not be representative of the true levels of time spent sedentary [94, 95]. Finally, specific environments (e.g., school) may reduce the between individual variability in sedentary time [96], and since young people spend most of their day at school, it is possible that accelerometer measurements during awake time will limit the possibility to detect associations with predicting factors. On the other hand, the ActivityStat hypothesis suggest that when physical activity is increased or decreased at one time, there will be a compensatory change at another time [97], so whether this issue has a large impact on the results is uncertain.”

The majority of the studies assessing sedentary behavior by self-report did not provide information about the validity and reliability of the measurement. Several of the identified studies included relatively small sample sizes and may not be adequately powered to identify weak, but true associations. The majority of the studies examined children aged 11 years or younger, and it is unknown whether the magnitude of the association between the examined predictors and sedentary behavior changes by age and may become apparent later in life. Another limitation is the reliability of prenatal factors such as birth weight. Several studies used data from birth records or parentally reported at birth, which should provide accurate measurements, however some studies assessed birth weight retrospectively from the parents, which may be prone to misclassification. Finally, our aim was to examine physical factors that may be causally associated with the outcome, rather than those correlated with sedentary behavior. The included studies examined prenatal, birth and infancy factors that precedes sedentary behavior later in life, and several of the included studies were prospective in design, thereby allowing determination of the direction of associations. However, an observational study design does not provide proof of causation per se. Additional observational studies employing the Bradford Hill criteria [96] when evaluating the results or randomization within a trial are warranted to determine causality.

### Future research

The research in this field is currently sparse, and the evidence whether prenatal, birth and early life factors are predictors of sedentary behavior is weak. There is a further need to understand whether associations develop through physical/mechanical pathways, for example accumulating adipose tissue might constrain physical movement; or through metabolic pathways, for example early adaptations in fuel metabolism might influence the availability for fuel utilization for physical activity at later ages. This applies not only to the development within a child, but also the intergenerational associations of maternal pregnancy physiology with offspring sedentary behavior. To increase our knowledge whether factors early in life influence not only health outcomes but also health-related behaviors such as sedentary behaviors and physical activity, including accurate and valid assessment of these behaviors or analyzing existing data in high quality birth cohorts are warranted. The effect sizes for any association between prenatal, birth and early life predictors and sedentary behavior appear small, and studies must be adequately powered enough to detect these modest, but perhaps important associations. Finally, although several potentially confounding factors have been included in existing studies, future studies may consider a wider range of both biological and socio-demographic confounders.

## Conclusion

The results from this systematic review suggest that heritability and early childhood BMI may be potential predictors for sedentary behavior in young people. No evidence was found for a relationship between birth weight and gestational age and later sedentary behavior. There is insufficient evidence whether other prenatal, birth and early life physical factors act as predictors of later sedentary behavior in young people.

## Abbreviations

BMI, body mass index; PROSPERO, the international prospective register of systematic reviews.

## Additional files

Additional file 1: Search strategy (Pubmed). (DOCX 14 kb) Additional file 2: Items included in the checklist for assessing the quality of the included studies. (DOC 22 kb)
