# Supplementary material for: Prenatal, birth and early life predictors of sedentary behavior in young people: a systematic review
Source: Int J Behav Nutr Phys Act. 2016 Jun 7;13:63. doi: 10.1186/s12966-016-0389-3 (PMC4897914; doi:10.1186/s12966-016-0389-3)
Supplement: Additional file 1: — Search strategy (Pubmed). (DOCX 14 kb) [file 12966_2016_389_MOESM1_ESM.docx]

**Additional file 1.** Search strategy (Pubmed)

| **Search terms** |
| --- |
| #1 (“Sedentary” OR “Sitting” OR “Physical inactivity” OR “Physically inactive” OR “Screen time” OR “Television” OR “TV” OR “Video game” OR “Video games” OR “Videogame” OR ”Videogames” OR “Video-game” OR “Video-games” OR “Gaming” OR “Computer” OR “Computer use” OR “Computer time” OR “Screen based entertainment” OR “Screen-based entertainment”)  AND  #2 (“Children” OR “Child” OR “Youth” OR “Youths” OR “Adult child” OR “Adult children” OR “Adolescent” OR “Adolescents” OR “Teenager” OR “Teenagers” OR “Teen” OR “Teens”) |
| **Search word #1 and #2 were combined with the other terms (#3-17) separately.** |
| #3 (“Maternal” AND (“Body weight” OR “Body weights” OR “Weight” OR “Body size” OR “Body sizes” OR “BMI” OR “Body mass index”)) |
| #4 (“Maternal” AND (“Age” OR “Ages” OR “Age group” OR “Age groups”)) |
| #5 ((“Maternal behaviour” OR “Maternal behaviours” OR “Maternal”) AND (“Sedentary” OR “Sitting” OR “Physical inactivity” OR “Physically inactive” OR “Screen time” OR “Television” OR “TV” OR “Video game” OR “Video games” OR “Videogame” OR “Videogames” OR “Video-game” OR “Video-games” OR “Gaming” OR “computer” OR “Computer use” OR “Computer time” OR “screen based entertainment” OR “screen-based entertainment”)) |
| #6 ((“Maternal behaviour” OR “Maternal behaviours” OR “Maternal”) AND (”Physical activity” OR “Physical activities” OR “Physically active” OR “Physical exercise” OR “Exercise” OR “Motor activity”)) |
| #7 (“Birth weight” OR “Birth weights” OR “Neonatal weight” OR “Newborn weight” OR “Postnatal weight”) |
| #8 (“Ponderal index” OR “Heel-crown length”) |
| #9 (“Temperament”) |
| #10 (“Weight gain” OR “Weight gains” OR “Growth” OR “Growths” OR “Growth trajectory” OR “Growth trajectories” OR “Body weight changes” OR “Body weight change” OR “body weight gain” OR “body weight gains” OR “Postnatal weight gain” OR “Catch-up growth”) |
| #11 (“Gestational age” OR “Gestational ages” OR “Gestational” OR “Maturity” OR “Fetal age” OR “Fetal ages” OR “Fetal maturity”) |
| #12 (“Child development” OR “Infant development” OR “Motor development” OR “Motor milestones” OR “Motor milestone” OR “Motor skills” OR “Motor skill” OR “Motor coordination” OR “Postnatal development”) |
| #13 (“heritability” OR “heritable” OR “genetic” OR “genetic association studies” OR “genetic phenomena” OR “gene” OR “twin-studies”) |
| #14 (“body weight” OR “BMI” OR “adiposity” OR “fat mass” OR “body mass index” OR “body length” OR “fat free mass” OR “body height” OR “body size” OR “waist circumference”) |
| #15 ((“Maternal behaviour” OR “Maternal behaviours” OR “Maternal”) AND ("tobacco smoking" OR "smoking"))  #16 "birth order"  #17 ("Multiple Pregnancy" OR "Multiple Pregnancies" OR "singleton pregnancies" OR " singleton pregnancy") |
