# Supplementary material for: Prenatal, birth and early life predictors of sedentary behavior in young people: a systematic review
Source: Int J Behav Nutr Phys Act. 2016 Jun 7;13:63. doi: 10.1186/s12966-016-0389-3 (PMC4897914; doi:10.1186/s12966-016-0389-3)
Supplement: Additional file 2: — Items included in the checklist for assessing the quality of the included studies. (DOC 22 kb) [file 12966_2016_389_MOESM2_ESM.doc]

**Additional file 2.** Items included in the checklist for assessing the quality of the included studies

1. Question/objective sufficiently described?
2. Study design evident and appropriate?
3. Method of subject/comparison group selection or source of information/input variables described and appropriate?
4. Subject (and comparison group, if applicable) characteristics sufficiently described?
5. Outcome and (if applicable) exposure measure(s) well defined and robust to measurement/misclassification bias? Means of assessment reported?
6. Sample size appropriate?
7. Analytic methods described/justified and appropriate?
8. Some estimate of variance is reported for the main results?
9. Controlled for confounding?
10. Results reported in sufficient detail?
11. Conclusions supported by the results?
